# Phase I/II study of erlotinib, carboplatin, pemetrexed, and bevacizumab in chemotherapy-naïve patients with advanced non-squamous non-small cell lung cancer harboring epidermal growth factor receptor mutation

**DOI:** 10.18632/genesandcancer.145

**Published:** 2017-05

**Authors:** Takayasu Kurata, Aya Nakaya, Takashi Yokoi, Maiko Niki, Kayoko Kibata, Yuki Takeyasu, Yoshitaro Torii, Yuichi Katashiba, Makoto Ogata, Takayuki Miyara, Shosaku Nomura

**Affiliations:** ^1^ First Department of Internal Medicine, Kansai Medical University, , Shin-machi, Hirakata, Osaka, Japan

**Keywords:** four-drug combination therapy, EGFR TKI, maintenance, phase I/II study, bevacizumab

## Abstract

**Background:**

Epidermal growth factor receptor tyrosine kinase inhibitors significantly prolong the progression-free survival of patients with non-squamous non-small cell lung cancer (NSCLC). However, most patients develop tumor regrowth and their prognosis remains poor. A new treatment strategy for NSCLC harboring EGFR mutation is therefore necessary.

**Methods:**

In phase I, eligible patients were administered oral erlotinib daily and intravenous pemetrexed, carboplatin, and bevacizumab every 3 weeks for four cycles with maintenance of pemetrexed and bevacizumab until progressive disease was observed. The dose of erlotinib was 100 mg for dose level 1 and 150 mg for dose level 2. The doses of pemetrexed, carboplatin, and bevacizumab were fixed at 500 mg/m^2^, area under the concentration–time curve of 6 mg/mL · min, and 15 mg/kg, respectively. The dose-limiting toxicities were grade 3/4 neutropenia with fever or infection, grade 4 leukopenia lasting for ≥7 days, grade 4 thrombocytopenia, grade 3/4 uncontrollable nonhematological toxicity, and delayed administration of the subsequent cycle by >2 weeks because of adverse events.

**Results:**

Six patients were enrolled in phase I (dose level 1, n = 3; dose level 2, n = 3). During the induction phase, grade 3 neutropenia without fever was observed in one patient at dose level 1 and two patients at dose level 2. Grade 3 anemia was reported in one patient at dose level 1 and grade 3 thrombocytopenia was reported in two patients at dose level 1 and dose level 2, respectively.

**Conclusion:**

Four-drug combination therapy is a feasible and promising.

## INTRODUCTION

Non-small cell lung cancer (NSCLC) accounts for about 80%–85% of all primary lung cancers. Most affected patients have metastatic disease, resulting in a poor prognosis. Chemotherapy plays an important role in the treatment of advanced NSCLC. The standard first-line chemotherapy is platinum-doublet combination therapy, which produces a modest overall survival benefit and quality of life. Since the discovery of activating epidermal growth factor receptor (EGFR) mutations and anaplastic lymphoma kinase rearrangements, patients harboring these mutations have been assigned to different subsets. Several large phase III studies have shown that EGFR tyrosine kinase inhibitors (EGFR TKIs; gefitinib, erlotinib, and afatinib) significantly prolong progression-free survival (PFS) compared with platinum doublet chemotherapy [1-6], and EGFR TKI monotherapy has thus been considered the standard first-line treatment for patients with TKI-sensitive EGFR mutations. Despite the efficacy of EGFR TKIs in such patients, the median PFS obtained by EGFR TKI therapy ranges from only 10 to 14 months, with most patients relapsing. After EGFR TKI failure, cytotoxic chemotherapy is currently considered the second-line treatment in clinical practice; however, this treatment remains associated with a poor prognosis. Therefore, there is a need for new therapeutic strategies to further improve overall survival among this patient population. One of the most promising strategies is the development of new agents, such as third-generation EGFR TKIs, with the aim of overcoming resistance arising from T790M mutation. A further challenge is the evaluation of combination therapy involving EGFR TKIs and bevacizumab or cytotoxic chemotherapy. Several clinical trials to evaluate combination treatments have already been performed. Seto et al. [7] conducted a randomized phase II study to compare the combination of erlotinib and bevacizumab versus erlotinib alone and reported a superior PFS in association with the combination therapy (median PFS was 16.0 months with erlotinib and bevacizumab and 9.7 months with erlotinib alone). The North East Japan Study Group and Tokyo Cooperative Oncology Group subsequently performed a randomized phase II study of concurrent versus sequential alternation of gefitinib and cytotoxic chemotherapy (carboplatin plus pemetrexed) [8]. The median PFS was 18.3 months for the concurrent regimen and 15.3 months for the sequentially alternating regimen, and the median survival times were 41.9 and 30.7 months, respectively. The authors selected the concurrent regimen as the investigational arm in a subsequent phase III study because of convenience, although both regimens showed promising efficacy. Although both were randomized phase II studies, combination therapy with an EGFR TKI and bevacizumab or platinum-doublet chemotherapy has been shown to be promising. Based on these results, we planned a combination phase I/II study to evaluate four-drug combination therapy involving an EGFR TKI, bevacizumab, and platinum-doublet chemotherapy. We first conducted the phase I part of the study to confirm the safety of the four-drug combination therapy and determine the appropriate dosages for phase II. The results of this phase I/II study are herein reported.

## RESULTS

Six patients (level 1, n = 3; level 2, n = 3) were enrolled in this study. The patients’ demographics are shown in Table 1. Five patients were female, and histological examination revealed adenocarcinoma in all patients. With respect to the EGFR mutation type, four patients had an exon 19 deletion and the remaining two had an exon 21 L858R mutation. All six patients could progress to the maintenance phase and so were evaluable for toxicity. The major AEs reported following the first course and all courses are listed in Table 3. In the DLT evaluation period, grade 3 neutropenia was observed in only one patient at dose level 1, and grade 3 thrombocytopenia, neutropenia, and stomatitis occurred in two, one, and one patient at dose level 2, respectively. These findings are not consistent with the DLT criteria; therefore, no DLT events were observed. In the evaluation of all courses, the most frequent AEs reported during the induction phase were hematological toxicities. During the induction phase, at dose level 1, grade 3 thrombocytopenia was seen in two patients, and grade 3 neutropenia and grade 3 anemia were seen in one patient. At dose level 2, grade 3 neutropenia and grade 3 thrombocytopenia were seen in two patients each. Unlike in the induction phase, hematological toxicities in the maintenance phase were generally mild. During the maintenance phase, at dose level 1, grade 3 anemia and grade 3 thrombocytopenia were each seen in two patients. At dose level 2, grade 3 anemia was seen in one patient. The most frequent non-hematological toxicity was appetite loss, which was reported in four patients at both levels during the induction phase. Stomatitis was reported in two patients in the induction phase and three patients in the maintenance phase. Grade 2 interstitial pneumonia occurred in one patient after the fifth course. No treatment-related deaths were observed. The details of the individual patients are shown in Table 2. According to the protocol criteria, because each of the two dose levels was tolerable, the recommended dose level for phase II was determined as erlotinib at 150 mg/day with carboplatin at an AUC of 6 mg/mL min, pemetrexed at 500 mg/m2, and bevacizumab at 15 mg/kg.

**Table 1:** Patient demographics

Variable	
Total number of patients(n)	6
Median age(years old)(range)	72 (46-76)
Sex(n)	
Male	1
Female	5
ECOG Performance Status(n)	
0	4
1	2
Smoking status(n)	
never	3
current	3
Histology(n)	
Adenocarcinoma	6
EGFR mutation type(n)	
exon 19 deletion	4
L858R in exon 21	2

ECOG: Eastern Cooperative Oncology Group EGFR: Epidermal growth factor receptor

**Table 2:** Patients’ characteristics

Case #	age (y/o)	sex	Histology	EGFR mutations	EML4-ALK	Brain metastases	Cycles of maintenance(n)	Dose level	Months on study	Status	Reason for withdraw
1	72	F	Adenocarcinoma	L858R(exon21)	N	N	18	1	32.1+	withdrew	AEs
2	46	F	Adenocarcinoma	deletion in exon 19	N	Y	15	1	14.7	withdrew	PD
3	74	F	Adenocarcinoma	deletion in exon 19	N	N	34	1	30.3+	going	-
4	71	F	Adenocarcinoma	deletion in exon 19	N	N	2	2	30.3+	withdrew	mental problem
5	76	M	Adenocarcinoma	deletion in exon 19	N	N	2	2	9.6	withdrew	AEs
6	71	F	Adenocarcinoma	L858R(exon21)	N	N	10	2	14.9	withdrew	PD

EGFR: Epidermal growth factor receptor EML4-ALK: echinoderm microtubule associated protein-like4 anaplastic lymphoma kinase AEs: adverse events PD: progressive disease

**Table 3:** Adverse events

Phase	Induction phase			Maintenance phase		
Level	Level 1	Level 2	total(n)	Level 1	Level 2	total(n)
Erlotinib Dose(mg)	100	150		100	150	
Neutropenia				-		-
Grade 2				1		1
Grade 3-4	1	2	3	-		-
Anemia				-		-
Grade 2	1	1	2	-		-
Grade 3-4	1		1	2	1	3
Thrombocytopenia				-		-
Grade 2						-
Grade 3-4	2	2	4	2		2
AST increased		-		-		-
Grade 2		-		-		-
Grade 3-4	1	-	1	1		1
ALT increased		-		-		-
Grade 2		-		-		-
Grade 3-4	1	-	1	1		1
T-bil increased		-		-		-
Grade 2		1	1	-		-
Grade 3-4		-		-		-
Fatigue		-		-		-
Grade 2	1	-	1	1		1
Grade 3-4		-		-		-
Stomatitis		-		-		-
Grade 2		-		1	1	2
Grade 3-4	1	1	2	1		1
Nausea		-		-		-
Grade 2		1	1	-		-
Grade 3-4		-		-		-
Appetite loss		-		-		-
Grade 2	2	2	4	1		1
Grade 3-4		-		-		-
Dysgeusia		-		-		-
Grade 2	1	-	1	-		-
Grade 3-4		-		-		-
Constipation		-		-		-
Grade 2		1	1	-		-
Grade 3-4		-		-		-
Diarrhea		-		-		-
Grade 2	1	-	1	-		-
Grade 3-4		-		-		-
Rash		-		-		-
Grade 2		-		-		-
Grade 3-4		-		1		1
Hypertension		-		-		-
Grade 2	1	-	1	-		-
Grade 3-4		-		-		-
Infection		-		-		-
Grade 2		1	1	-	2	2
Grade 3-4		-		-		-
Alopecia		-		-		-
Grade 2		1	1	-		-
Grade 3-4		-		-		-
Bradycardia		-		-		-
Grade 2		-		-	1	1
Grade 3-4		-		-		-
Fever		-		-		-
Grade 2		-		-	1	1
Grade 3-4		-		-		-
Interstitial Lung Disease		-		-		-
Grade 2		-		-	1	1
Grade 3-4	-	-	-	-	-	-

AST: aspartate transaminase ALT: alanine transaminase

All six patients were also assessable for treatment response, and all had a partial response for an overall response rate of 100%. At the time of writing, three of the six patients had not yet developed PD. At dose level 1, one patient developed PD during the first 14.7 months after registration and one patient withdrew from the study because of AEs during the first 17.9 months after registration; however, this patient maintained a partial response after cessation of the study (PFS, 32.1 months+). The remaining patient was still undergoing treatment at the time of writing (PFS, 30.3 months+). All three patients at dose level 2 withdrew from the study. One patient withdrew because of a mental condition at 3.7 months; however, she still showed a partial response (PFS, 30.3 months+). The other two patients developed PD (PFS, 9.6 and 14.6 months, respectively); one withdrew because of an AE but developed PD soon after cessation of the study.

The four patients with an exon 19 deletion had a PFS of 14.7 months, 30.3 months+, 30.3 months+, and 9.6 months, respectively. The two patients with an exon 21 L858R mutation had a PFS of 32.1 months+ and 14.9 months, respectively.

## DISCUSSION

Although EGFR TKI monotherapy is considered the standard first-line treatment for patients with TKI-sensitive EGFR mutations, most patients develop recurrence within approximately 1 year. Combination therapies involving EGFR TKIs have recently been evaluated to improve prognosis. Regimens involving erlotinib and bevacizumab [7]; gefitinib and pemetrexed [11]; and gefitinib, carboplatin, and pemetrexed [8] have shown promising results, although these combinations were evaluated only in randomized phase II studies. We plan to examine the efficacy of a combination of all four of these agents in a phase II study to further assess and potentially increase the strength of combination therapy. An advantage of this combination therapy is that the toxicity profiles of EGFR TKIs and carboplatin, pemetrexed, and bevacizumab do not appear to overlap. Prior to evaluating the effect of this four-drug combination therapy, we performed the present phase I study to examine the safety of this combination and determine the appropriate doses. The most frequent toxicities associated with this four-drug combination therapy were hematological toxicities, which may have been caused by the cytotoxic chemotherapy agents, and skin toxicities caused by the EGFR TKIs. Because the frequency and severity of the hematological toxicities were similar to those observed in our previous phase II study, which was performed to evaluate the efficacy and safety of carboplatin, pemetrexed, and bevacizumab [12], we consider that this combination is manageable and tolerable despite the small sample size assessed. In fact, all patients were able to progress to maintenance therapy, although some experienced a treatment delay and dose reduction because of toxicities in the induction phase. However, although more than 10 courses of maintenance therapy were administered at dose level 1, two patients at this level refused to continue with maintenance therapy and one patient at dose level 2 withdrew from the study because of interstitial pneumonia. Based on the evaluation of tolerability throughout this study and in accordance with the protocol criteria, we consider that the combination of these drugs is safe at the recommended doses (150 mg/day for erlotinib, AUC of 6 mg/mL · min for carboplatin, 500 mg/m2 for pemetrexed, and 15 mg/kg for bevacizumab).

With respect to the efficacy of this four-drug combination therapy, all six patients had partial responses. At dose level 1, all patients were able to continue maintenance therapy for >10 cycles. The longer the period of maintenance therapy, the better the prognosis. Compared with the currently established median PFS of EGFR TKI-based combination therapies (16.0 months for erlotinib and bevacizumab [7]; 18.3 months for gefitinib, carboplatin, and pemetrexed [8]; and 15.8 months for gefitinib and pemetrexed [11]), the PFS observed in the present study is promising.

Our study has a limitation. EGFR TKIs are key drugs for patients with EGFR mutation-positive cancer. In the present study, however, the dose escalation was performed for erlotinib. Thus, patients who received 100 mg of erlotinib might not have received the maximal benefit of the EGFR TKIs. No DLT events were observed during the DLT evaluation period; thus, the recommended dose of erlotinib is 150 mg/day. However, some AEs associated with erlotinib occur as late-onset events. As a consequence, dose level 1 patients were able to continue with >10 cycles of maintenance therapy because low-dose erlotinib is more manageable for long-term AEs.

A recent phase III study revealed no difference in PFS between gefitinib (250 mg/day) and erlotinib (150 mg/day) in patients with previously treated advanced adenocarcinoma [13]. The dose of gefitinib was one-third of the MTD, whereas the dose of erlotinib was equal to the MTD. Thus, the effect of erlotinib might not differ between the 100 and 150 mg doses.

To evaluate the four-drug combination regimen, we will next proceed with a multicenter phase II study. Some treatment regimens have recently shown different efficacies between patients with an exon 19 deletion and those with an exon 21 L858R mutation. Additionally, the effect of this treatment did not differ between patients with these two mutations.

In conclusion, the four-drug combination therapy (induction with erlotinib, carboplatin, pemetrexed, and bevacizumab followed by maintenance with erlotinib, pemetrexed, and bevacizumab) is feasible and promising. No DLTs were observed, and the recommended dose was determined to be 150 mg/day for erlotinib with an AUC of 6 mg/mL · min for carboplatin, 500 mg/m2 for pemetrexed, and 15 mg/kg for bevacizumab every 3 weeks.

## METHODS

### Study Design and Patients

This combination phase I/II study was designed to evaluate the efficacy and safety of four-drug combination therapy in patients with previously untreated non-squamous NSCLC harboring EGFR mutation. Phase I was carried out in a single facility, Kansai Medical University Hospital, Osaka, Japan. Patients with histologically or cytologically confirmed advanced non-squamous NSCLC who had received no previous chemotherapy were eligible. The eligibility criteria were as follows: (1) age ≥20 years, (2) measurable lesions; (3) Eastern Cooperative Oncology Group performance status (PS) of 0 or 1; (4) clinical stage IIIB or IV cancer or postoperative recurrence; (5) tumor with an EGFR mutation other than T790M in exon 20; (6) chemotherapy naivety (neoadjuvant or adjuvant chemotherapy was allowed if ≥6 months had passed since completion of neoadjuvant or adjuvant chemotherapy); (7) life expectancy of ≥90 days; and (8) adequate organ function (absolute neutrophil count ≥1500/mm3, platelet count ≥100,000/mm3, hemoglobin concentration ≥9.0 g/dL, serum total bilirubin concentration ≤1.5 mg/dL, serum transaminase concentration ≤2.5 times the upper normal limit, serum creatinine concentration ≤1.5 times the upper normal limit, SpO2 ≥90% on room air, and proteinuria ≤1+). The exclusion criteria were as follows: (1) pulmonary fibrosis or interstitial pneumonitis with apparent abnormalities on chest X-ray examination; (2) history of radiation for treatment of the primary tumor of the chest; (3) history of gross hemoptysis (≥2.5 mL) or continuous history of bloody sputum; (4) high likelihood of complications related to hemorrhage (coagulation disorders, obvious tumor infiltration into the large blood vessels, obvious tumor cavity on imaging examination, or obvious tumor invasion into the bronchial mucosa by imaging or endoscopy); (5) severe infection or severe comorbidities such as bleeding peptic ulcer, ileus, heart failure, renal failure, or poorly controlled diabetes; (6) massive pleural effusion or ascites; (7) active concurrent malignancies; and (8) severe drug allergies. This study was approved by the Institutional Review Board of Kansai Medical University (Osaka, Japan) and was conducted in accordance with the Declaration of Helsinki. Written informed consent was obtained from all patients.

### Pretreatment and Follow-up Studies

As a pretreatment assessment, a complete history was obtained including sex, age, height, weight, PS, smoking history, histologic diagnosis, tumor stage, type of EGFR mutation, previous treatment, imaging assessment, and presence of complications. The pretreatment laboratory investigation included a complete blood cell count, differential white blood cell count, platelet count, serum electrolyte concentrations, total bilirubin concentration, transaminase concentration, alkaline phosphatase concentration, lactate dehydrogenase concentration, blood urea nitrogen concentration, creatinine concentration, SpO2, and electrocardiogram findings. After initiation of therapy, the blood count, blood chemistry, and chest X-ray examinations were repeated once within 3–4 weeks. Lesions were measured once within 6–8 weeks. Toxicity was evaluated in accordance with the National Cancer Institute Common Terminology Criteria for Adverse Events (version 4.0) [9]. Tumor responses were assessed using the Response Evaluation Criteria in Solid Tumors (version 1.1). [10] The time to progression was measured from the date of registration to the date of first progression or death of any cause. The survival time was measured from the date of registration to the date of death or latest follow-up.

### Treatment and Dose Escalation

All eligible patients were intravenously administered fixed doses of pemetrexed at 500 mg/m2, carboplatin at an area under the concentration–time curve (AUC) of 6.0 mg/mL min, and bevacizumab at 15 mg/kg on day 1 every 3 weeks for four cycles. After four cycles had been completed, maintenance pemetrexed and bevacizumab were continued until progressive disease (PD) was observed. During the administration of pemetrexed, all patients received supplemental folic acid and vitamin B12. Erlotinib was taken as a daily oral dose; the initial dose was 100 mg/day and was increased to 150 mg/day in the absence of dose-limiting toxicities (DLTs). If DLTs occurred at dose level 1, the dose level of erlotinib was reduced (level 0, 50 mg/day). At least three patients were treated at each dose level, and three additional patients were included if a DLT was observed. The maximum tolerated dose (MTD) was defined as the dose level at which the first three patients, or three of any six patients, all experienced DLTs. The following treatment-related adverse events (AEs) were defined as DLTs: grade ≥3 febrile neutropenia, grade ≥3 neutropenia with infection, grade 4 neutropenia or leukopenia for >7 days, grade 4 thrombocytopenia, grade ≥3 non-hematological toxicities despite appropriate treatment, and delayed administration of the subsequent course by >2 weeks. If DLTs occurred during the previous treatment course and additional antitumor effects could be expected, the doses of carboplatin and pemetrexed could be decreased twice (carboplatin: AUC, 5.0 and 4.5 mg/mL · min; pemetrexed: 400 and 300 mg/m2). If grade ≥2 thromboembolism, grade ≥3 bleeding, myocardial infarction, arrhythmia, or perforation of the digestive tract occurred, carboplatin, pemetrexed, and bevacizumab were discontinued. In cases of severe bevacizumab- or erlotinib-related toxicities, bevacizumab or erlotinib were temporarily or permanently suspended. When these agents were subsequently restarted, while the dose of bevacizumab continued unchanged, that of erlotinib could be reduced according to the physician's judgment
